# An Effective Framework for Deep-Learning-Enhanced Quantitative Microwave Imaging and Its Potential for Medical Applications

**DOI:** 10.3390/s23020643

**Published:** 2023-01-06

**Authors:** Álvaro Yago Ruiz, Marta Cavagnaro, Lorenzo Crocco

**Affiliations:** 1 Department of Information Engineering, Electronics, and Telecommunications, University of Rome “La Sapienza”, 00184 Rome, Italy; 2 CNR-IREA National Research Council of Italy, Institute for Electromagnetic Sensing of the Environment, 80124 Napoli, Italy

**Keywords:** microwave imaging, deep learning, inverse scattering, orthogonality sampling method, U-Net, convolutional neural network

## Abstract

Microwave imaging is emerging as an alternative modality to conventional medical diagnostics technologies. However, its adoption is hindered by the intrinsic difficulties faced in the solution of the underlying inverse scattering problem, namely non-linearity and ill-posedness. In this paper, an innovative approach for a reliable and automated solution of the inverse scattering problem is presented, which combines a qualitative imaging technique and deep learning in a two-step framework. In the first step, the orthogonality sampling method is employed to process measurements of the scattered field into an image, which explicitly provides an estimate of the targets shapes and implicitly encodes information in their contrast values. In the second step, the images obtained in the previous step are fed into a neural network (U-Net), whose duty is retrieving the exact shape of the target and its contrast value. This task is cast as an image segmentation one, where each pixel is classified into a discrete set of permittivity values within a given range. The use of a reduced number of possible permittivities facilitates the training stage by limiting its scope. The approach was tested with synthetic data and validated with experimental data taken from the Fresnel database to allow a fair comparison with the literature. Finally, its potential for biomedical imaging is demonstrated with a numerical example related to microwave brain stroke diagnosis.

## 1. Introduction

In the last few years, microwave imaging (MWI) has emerged as a candidate technology to perform medical diagnostics, being non-invasive, non-harmful, low-cost, and portable [1,2]. Medical MWI is enabled by the heterogeneous dielectric properties (relative permittivity and conductivity) that different human tissues exhibit at microwave frequencies, based on their typology and on their physio-pathological status [3]. More precisely, MWI has been mainly considered for breast cancer screening [4], the diagnosis of cerebrovascular diseases [1], treatment monitoring [5,6,7,8,9], and, more recently, Alzheimer’s disease monitoring [10].

Despite the growing interest, one of the factors hampering the widespread adoption of MWI in the clinical sphere is the need for solving an inverse scattering problem (ISP) to obtain the relevant diagnostic information (i.e., the dielectric properties of the inspected tissue). ISPs belong to the class of non-linear and ill-posed inverse problems [11], whose solution may lead to unreliable diagnostic outcomes, if not properly tackled.

Coping with these difficulties calls for a methodological effort aimed at developing reliable solution strategies for the ISP. In this respect, deep learning (DL) is rising as a powerful tool to provide accurate and reliable solutions for ISPs in a computationally efficient way [12,13,14].

Several DL approaches to ISPs have been proposed in the literature. Among them, *physics-assisted* approaches have attracted remarkable attention [13]. In these approaches, domain knowledge on wave scattering physics is incorporated into the input or in the internal architecture of the neural network, having a beneficial effect on its performance [15,16]. In particular, embedding domain knowledge by pre-processing the MWI scattering measurements with a traditional imaging algorithm is a convenient strategy, since the DL architecture does not need to learn the physics behind the transformation from the measurement domain to the imaging domain. This circumstance results in a simpler problem and, consequently, a less-demanding training of the DL model. For instance, Wei et al. [17] provided a comparison of training a U-Net [18] using scattering measurements as the inputs versus using images, depicting an estimation of the corresponding currents induced in the imaging domain. The comparison shows the superior performance of aiding the training with an MWI algorithm instead of directly training with the scattering measurements. Similar outcomes were obtained in [19], where a rough image obtained via backpropagation was fed into a generative adversarial network trained to retrieve the salient high-level features of the unknown targets. Moreover, in [20], a U-Net was employed to refine an image depicting a preliminary coarse guess of the permittivity based on the Born approximation.

In [21], the authors proposed a physics-assisted approach in which a U-Net was trained to transform images obtained via an MWI technique, the orthogonality sampling method (OSM) [22,23], into a binary image for a reliable, user-independent, and real-time retrieval of the shape of the unknown targets. However, this approach could not retrieve the electromagnetic properties of the targets, thus having limited applicability for medical diagnostic purposes. To overcome this limitation without resorting to a different MWI algorithm, it is possible to exploit a distinctive characteristic of the OSM. Besides providing an estimate of the shape of the targets, the OSM images also embed qualitative information on the relative variation of their dielectric properties with respect to the host medium [23]. However, such information on the contrast is encoded in a subtle way, so that retrieving the contrast would require facing another inverse problem [23].

In this paper, the problem of retrieving the morphological and electromagnetic properties of unknown targets encoded in the OSM images is cast in terms of a segmentation task performed by a U-Net architecture. In particular, the U-Net is trained to associate the pixels of the OSM imaging results with a finite set of contrast values. In doing so, the training stage is remarkably simplified, as the network will just need to learn how to perform in a robust way the OSM image contrast association within the given interval. Moreover, as the framework relies on the OSM to supply the domain knowledge to the U-Net, it is able to deliver real-time automated results. Finally, such an approach is well matched with medical MWI scenarios, wherein each tissue in the interested portion of the body can be effectively described by an average complex permittivity [24].

It is worth remarking that, since the OSM imaging results encode the information on the contrast in a non-obvious way, the adoption of standard segmentation algorithms (based on, e.g., thresholding) is ineffective, as it would require a priori assumptions on the relationship between the quantitative changes in the OSM images and the contrast variations. Conversely, the capability of supervised machine learning to build models to tackle complex regression tasks provides the appropriate tool to perform this task efficiently. In this respect, the choice of U-Net is due to the fact that this neural network architecture was designed primarily for image segmentation [18], so that it can be conveniently exploited to prove the feasibility of the proposed approach.

Considering the positioning of this work in the relevant literature, this is the first time in which, to the best of our knowledge, the inverse scattering problem is approached in terms of a segmentation problem. Limiting the number of admissible contrast values to a discrete set was exploited in [25] for breast MWI, but there are some fundamental differences between [25] and the approach proposed herein. The authors of [25] presented a segmentation technique to partition microwave images achieved by previously solving the ISP. Since the ill-posedness and the non-linearity of the problem significantly impact the MWI results, the goal of the technique proposed in [25] was indeed to facilitate the evaluation of the image quality by segmenting it. To this end, the authors combined an unsupervised machine learning method with statistical techniques. Here, a computational framework to *solve* the ISP using the concept of segmentation is instead proposed. This original methodology was implemented using a purely supervised machine learning approach. In doing so, the network is fed an input that is repeatable and reliable, since the OSM image is formed without explicitly solving the ISP, but processing the MWI raw data by means of direct computations (scalar products) [23]. Hence, the U-Net in charge of the segmentation has an active role in solving the ISP and is not a post-processing tool.

In the following, the proposed DL-MWI framework is presented in the canonical 2D scalar problem (TM polarized fields) in free space and validated with experimental data taken from the Fresnel institute database [26], which represents the commonly adopted benchmark to assess MWI algorithms. The potential of the approach for biomedical imaging is then shown by means of a numerical example related to brain stroke diagnosis.

The rest of the paper is organized as follows. In Section 2, the ISP is formulated. The OSM is described in Section 3. In Section 4, the core of the approach is discussed, and its implementation to tackle the canonical ISP at hand is given in Section 5. Section 6 and Section 7 provide the performance assessment on simulated and experimental data, respectively. Section 8 presents the example dealing with brain stroke imaging. Conclusions follow. Throughout the paper, a time-harmonic behavior was supposed and the corresponding time factor $e^{j\omega t}$ assumed and dropped.

## 2. Problem Formulation

Let Ω denote the imaging domain, which hosts the cross-section Σ of a collection of targets invariant along one direction (say the *z*-axis). The domain Ω is embedded in a background medium, which, for the sake of simplicity, but without loss of generality, is assumed to be homogeneous and lossless; let $\varepsilon_{b}$ denote the relative permittivity of the background medium. Each target is characterized by a relative dielectric permittivity ε(r) and an electric conductivity σ(r), with r=(x,y). All materials are supposed to be non-magnetic, i.e., the magnetic permeability is everywhere that of vacuum $\mu_{0}$.

A multi-frequency multi-static measurement configuration is adopted to probe the targets. For each frequency *f* belonging to the set of $N_{f}$ frequencies used for the imaging experiment, the incident fields $E_{inc}$ are radiated by $N_{v}$ antennas located in $r_{t}\in \Gamma$, with $v=1,\ldots ,N_{v}$ and Γ being a closed curve located in the far-zone of Ω. For each transmitter, the interaction between the incident field and the targets gives rise to the scattered field $E_{s}$. The superposition of these two fields becomes the total field $E=E_{inc}+E_{s}$, which is measured by a set of $N_{m}$ receivers, which, without any loss of generality, are assumed to be located on Γ as well, with the receiver position being $r_{m}$ with $m=1,\ldots ,N_{m}$.

Given the frequency *f* and the pair of Tx–Rx antennas, the overall phenomenon is cast through a Fredholm-type integral equation as:

(1) $$E_{s}\left(r_{m},r^{\prime }\right)=k_{0}^{2}\int_{\Omega }G\left(r_{m},r^{\prime }\right)\tau \left(r^{\prime }\right)E\left(r^{\prime },r_{t}\right)dr^{\prime }$$

where $G(r_{m},r^{\prime })$ is the Green’s function of the assumed homogeneous background medium, $k_{0}=\omega \sqrt{\mu_{0}\varepsilon_{0}}$ is the wave number in free space at the working frequency, and $\tau \left(r\right)=\varepsilon_{eq}\left(r\right)/\varepsilon_{b}-1$ is the contrast function encoding the properties of the targets. $\varepsilon_{eq}\left(r\right)=\varepsilon \left(r\right)-j\sigma \left(r\right)/\omega \varepsilon_{0}$ denotes the relative complex permittivity of the targets, with *j* being the imaginary unit, ω=2πf the angular frequency, and $\varepsilon_{0}$ the dielectric permittivity of the vacuum.

The total field *E* is defined through another Fredholm integral equation of the first kind as:

(2) $$E\left(r,r_{t}\right)=E_{inc}\left(r,r_{t}\right)+k_{0}^{2}\int_{\Omega }G\left(r,r^{\prime }\right)W\left(r^{\prime },r_{t}\right)dr^{\prime }$$

where $W\left(r^{\prime },r_{t}\right)=\tau \left(r^{\prime }\right)E\left(r^{\prime },r_{t}\right)$ is the contrast source.

The retrieval of the contrast function τ from measurements of the fields they scatter is the objective of the ISP. However, due to the smoothing kernel of (1) and the dependence of the total field on τ, the problem turns out to be non-linear and ill-posed.

## 3. Orthogonality Sampling Method

The OSM provides an estimate of the unknown targets’ shape through the plot of an indicator function over the imaging domain. Such an indicator attains its higher values at points that are expected to belong to the targets [22].

For a given frequency belonging to the set of frequencies adopted for the imaging experiment, the OSM indicator *I* is defined as:

(3) $$I\left(r_{p}\right)=\left|\right|E_{red}\left(r_{p},r_{t}\right){\left|\right|}_{\Gamma }^{2},$$

where |||| denotes the $L^{2}$-norm computed on Γ, $r_{p}$ is a point of an arbitrary grid sampling the imaging domain Ω, and $E_{red}$ is the *reduced field*, which is computed as:

(4) $$E_{red}\left(r_{p},r_{t}\right)=<E_{s}\left(r_{m},r_{t}\right),G\left(r_{m},r_{p}\right){>}_{\Gamma }$$

where <,> denotes the scalar product on Γ.

In [23,27], it was shown that the reduced field is related to the contrast source *W* as

(5) $$E_{red}\left(r_{p},r_{t}\right)=\beta \int_{\Omega }J_{0}(k_{0}|r^{\prime }-r_{p}\left|\right)W\left(r^{\prime },r_{t}\right)dr^{\prime }$$

where $J_{0}$ is the first kind Bessel function of zero order and β is a constant.

Since the contrast sources *W* are null outside of the targets, (5) shows that the OSM indicator allows retrieving their shape, (3) being related to the superposition of the square amplitude of the contrast sources at hand. In addition to this, and more important for our purposes in this work, since *W* is directly related to the contrast distribution, from (5), it also follows that the spatial distribution of the OSM indicator locally depends on the electromagnetic properties of the investigated objects. In particular, in the case of piecewise homogeneous targets, *W* will exhibit discontinuities when the target properties abruptly change, and its values across the boundary will mainly depend on the contrast values. In fact, in the 2D TM case at hand, the electric field will be continuous across those boundaries. Accordingly, in the neighborhood of any discontinuity, also the OSM indicator will be characterized by larger relative values at those points corresponding to higher contrast values.

## 4. Contrast Retrieval via Deep Learning Segmentation of OSM Imaging Results

As discussed above, the OSM indicator map can be used to provide not only an estimate of the shape of the targets, but also a qualitative image of the behavior of the contrast τ in the imaging domain. However, quantifying the value of the contrast is not straightforward, since the relationship between the indicator value and the corresponding contrast is not obvious; see (5).

To cope with this difficulty, it is possible to exploit DL approaches, taking advantage of their known capability of tackling complex regression problems and outperforming traditional methods. In the case at hand, the regression problem would consist of associating with each pixel of the OSM indicator map a contrast value, to build a quantitative image of the EM properties of the targets.

Considering that the OSM map encodes the relative contrast variation, the approach that is herein proposed is to cast the regression problem at hand as a segmentation task. In so doing, the contrast τ to be retrieved is restricted to a limited number, say $N_{c}$, of possible values (segmentation classes) within a given interval, instead of belonging to a continuous range.

The segmentation classes can be selected in two ways. The first one consists of setting a range of τ values for a given problem and defining the number of classes accordingly. This approach requires the contrast range to be known a priori, while the number of classes is to some extent arbitrary, and its choice is mainly dictated by a tradeoff between a finer discretization of τ and the computational complexity (A larger number of classes entails the need for a larger training set.). The second option is application-specific and consists of matching the number of structures with different properties appearing in the imaging domain relevant to the specific application at hand. This, for instance, is the case of medical applications, where the different electromagnetic properties corresponding to the different tissues of interest have to be matched. As the first approach is more general, it will be adopted to describe the proposed framework and validate its performance. The second approach is instead adopted in Section 8, wherein the proposed framework is exploited in the specific case of brain stroke imaging.

**Figure 1:**
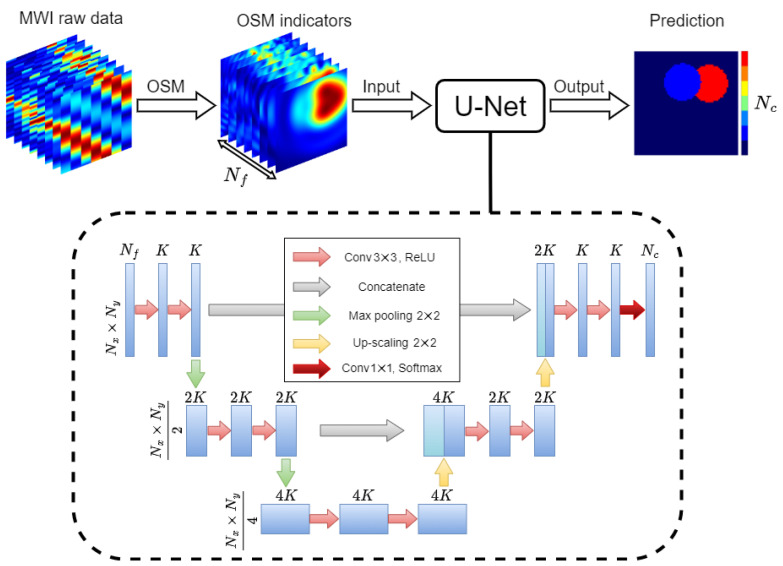
Flow diagram of the DL-MWI framework. The internal architecture of the U-Net with its different operations is shown inside the dashed line box.

Figure 1 describes the flow of the proposed approach: the raw data are processed via the OSM, and the obtained imaging results are fed into the DL model in charge of predicting the segmented contrast map. To build the DL model, a U-Net CNN [18] was exploited. This kind of neural network is a powerful architecture suitable to process data with a high spatial correlation, such as images [25], and allows its output to be retrieved as an image as well. In particular, a U-Net similar to the one in [17] was implemented. The structure of the network is shown in the lower part of Figure 1, wherein the operations the U-Net internally carries out are also indicated (see [18] for details).

The task performed by the U-Net can be cast as:

(6) $$\hat{T}=F_{\theta }\left(I\right)$$

where $F_{\theta }$ is the DL model, θ denotes the network parameters to be optimized in the training, $I\in R^{N_{p}\times N_{f}}$ is the input of the network given by the array of OSM indicator images obtained in the pre-processing step, and $\hat{T}\in R^{N_{p}\times N_{c}}$ is the segmented contrast map predicted by the network. $N_{p}=N_{x}\times N_{y}$ is the number of pixels into which the imaging domain is discretized to compute the OSM indicators, and $N_{f}$ is the number of frequencies adopted in the MWI experiment. It is worth noting that the exploitation of frequency diversity is crucial. As a matter of fact, OSM indicator maps are first normalized and, then, fed into the U-Net. As such, targets having the same shape and position, but with different electromagnetic properties, may be indistinguishable if a single image (either achieved with monochromatic data or merging information across the frequencies into a single OSM indicator) is supplied to the network. Instead, feeding the U-Net with an array of maps introduces a diversity in the input data, which allows the network to compare the information across frequencies and learn how to properly retrieve the contrast value.

To determine the optimal set of parameters of the U-Net, say $\hat{\theta }$, a supervised learning approach was adopted. To this end, a set of *N* contrast functions $\tau^{n}$ is exploited to build the training pairs $\left(T^{n},I^{n}\right)$, with n=1,…,N, where $T^{n}\in R^{N_{p}\times N_{c}}$ is the segmented map of $\tau^{n}$ and $I^{n}$ is the array of OSM maps obtained from the scattered field data corresponding to $\tau^{n}$. The optimal parameters distribution $\hat{\theta }$ is chosen by minimizing the categorical cross-entropy [28]. This minimization is performed iteratively by sequentially scanning the training set *P* times, where *P* is referred to as the number of epochs. For the *n*-th sample, the corresponding estimate of the optimal distribution, say ${\hat{\theta }}^{n}$, is defined as:

(7) $${\hat{\theta }}^{n}=\underset{\theta }{\arg\min}\left[-\sum_{i=1}^{N_{p}}\sum_{c=1}^{N_{c}}T_{i,c}^{n}\cdot log\;\;{\hat{T}}_{i,c}^{n}\right]$$

where ${\hat{T}}_{i,c}^{n}$ is the segmented contrast map predicted by the U-Net using the parameter distribution at the previous iteration. Finally, the optimal distribution is achieved after PN iterations (${\hat{\theta }}^{PN}$).

Once the training has been successfully carried out, the framework is ready to process new data. Notably, such an operation is carried out in real-time and without human supervision, since the OSM does not need to explicitly determine any regularization parameter to provide the $N_{f}$ indicator images and since both the OSM and the optimized network architecture $F_{\hat{\theta }}$ work in real-time.

## 5. Implementation of the DL-MWI Framework for the 2D ISP in Free Space

In this section, the implementation of the proposed DL-MWI framework to deal with the 2D canonical ISP in free space is presented.

Similar to [17,21], the adopted training set is made of a collection of lossless circular cylinders with a variable size, location, and permittivity. Note the lossless targets assumption does not entail a loss of generality and allows us to show that the trained framework can successfully process slightly lossy targets without retraining; see Section 7. Each sample of the training set consists of two, possibly overlapping cylinders, whose permittivities were selected to cover a wide range of contrast values, not necessarily limited to Born-like approximations. Furthermore, no profile was allowed to be partially outside of the imaging domain. More details of the setup conditions are listed in Table 1. The considered contrast range was $\left[0,2.5\right]$, which was divided into $N_{c}=7$ classes, as specified in the first two columns of Table 2.

For the training, the U-Net is fed by a pair made by the segmented ground truth and the corresponding OSM images. To build these pairs, the first step is to simulate the data corresponding to the targets constituting the training set, and the forward scattering problem has to be numerically solved. An approach based on the method of moments [29] was exploited, and the resulting convex optimization problem was solved by means of a conjugate gradient method made efficient by the use of the fast Fourier transform. This approach for the solution of the forward problem was implemented in a proprietary code (tested against analytical solutions) and applied to compute the field scattered by each target in the training set considering a multi-frequency multi-static configuration in which the curve Γ where the antennas are located is a circumference. The details of the adopted configuration, including the number of transmitters $N_{v}$, the number of receivers $N_{m}$, and the number of frequencies $N_{f}$, are reported in Table 1. Note the angular spacing for transmitters and receivers is the same as the one used in the Fresnel database [26,30], which is used in Section 7 to validate the proposed approach.

Previous work on the effect of corrupting $E_{s}$ with noise prior to computing the OSM indicators confirmed the robustness of the framework against noise thanks to the filtering effect of the OSM [21]. Therefore, noiseless data were used in the training stage and processed with the OSM. The resulting OSM indicators $I^{n}$ were calculated defining $N_{x}=N_{y}=64$ and $N_{f}=8$. A [0,1] normalization was carried out for each indicator before feeding them into the network [31].

Regarding $T^{n}$, ground truth segmented contrast images were obtained from the thresholding of the simulated targets, with $N_{x}=N_{y}=64$ and $N_{c}=7$. As shown in Table 2, each class was defined as the range of τ, whose values fall between two thresholds, making six classes for the targets and one for the background medium.

As far as the size of the training set is concerned, considering the positive results obtained in [21] using a 2000-sample training set for binary classification, a similar rule was adopted herein to set the size *N* of the training set. Accordingly, the number $N_{c}$ of segmentation classes dictated the size of the training set, which was assumed to be $N=N_{c}\times 1000$. Consequently, a total set of N=7000 scattering experiments involving lossless homogeneous circular cylinders were simulated. Among them, 90% were used as the training set and 10% as test set. The selection of the samples belonging to either set was performed using a 10-fold cross-validation scheme, leading to 10 experiments with varying training and test sets to be used in the performance evaluation, as detailed in Section 6.

**Figure 2:**
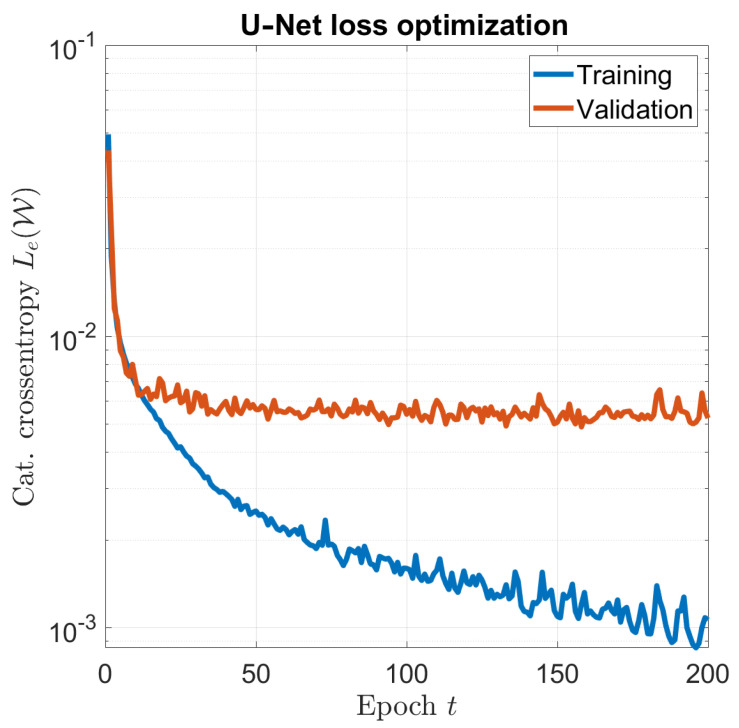
U–Net optimization: training and validation convergence.

The optimization of the network was carried out using the Adam optimizer [32], with a learning rate of $10^{-4}$. To improve convergence, a common strategy for the training is using a batch of samples $N_{b}$ in (7) instead of a single sample to obtain the optimal distribution in each iteration. In this work, $N_{b}=16$. The optimal solution was found after p=200. The overall training time was about 1 hour on a dual-GPU workstation, and Figure 2 shows the training and validation convergence curves.

## 6. Assessment of Proposed Framework: Simulated Data

The proposed DL-MWI framework as trained and optimized in the previous section was assessed with simulated data taken from the test cases excluded from the training. Accordingly, for each fold, the 700 samples excluded from the training were fed into the network, and the corresponding predictions were retrieved.

### 6.1. Performance Evaluation Metrics

The performance of the optimized model were quantitatively evaluated using two metrics. The first considered metric is the *Dice similarity coefficient* (DCS) [33], which is defined as:

(8) $$DSC=\frac{2\cdot TP}{2\cdot TP+FP+FN}$$

where TP, TN, FP, and FN stand for the count of true positives, true negatives, false positives and false negatives, respectively. In the problem at hand, TP was considered to be the count of all pixels correctly labeled in the corresponding segmented class. Conversely, TN was considered the count of all pixels correctly labeled as not belonging to a specific segmented class.

Although, as shown in [21], the DSC provides a more robust performance score than other metrics such as, e.g., accuracy, it may overestimate the performance in segmentation problems, if not used carefully. In particular, when a class has a proportion of pixels superior to the rest of the classes (typically the background), the DSC may provide an inflated performance score. This is because in those situations, the TP and FP outnumber the FN. The double contribution of the TP to the DSC worsens the estimation further [34]. Therefore, for the sake of comparison, the second metric called *Matthews correlation coefficient* (MCC) [35] was calculated as well:

(9) $$MCC=\frac{TP\cdot TN-FP\cdot FN}{\sqrt{DM}}$$

where DM=(TP+FP)(TP+FN)(TN+FP)(TN+FN).

### 6.2. Results

For each sample, the considered metrics, reported in Table 2, were calculated and averaged over the 10 folds. Overall, high performance scores were achieved in terms of the DSC for all contrast ranges. Regarding the MCC, performance scores were inferior to their DSC counterparts, as expected. Additionally, the MCC scores showed a decreasing pattern for increasing contrast values. This is possibly due to the increasing non-linear behavior of the ISP with higher contrast values [36].

**Figure 3:**
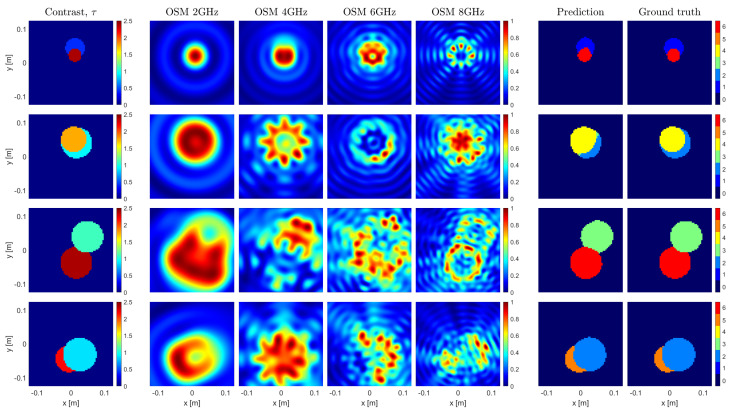
Imaging results of 4 test samples. One sample per row, with the first column representing the targets with their dielectric properties. The following 4 columns show 4 out of 8 single-frequency OSM indicators at 2, 4, 6, and 8 GHz. The 6th and 7th columns depict the segmented contrast prediction made by the U–Net and the ground truth segmented contrast map, respectively.

An example of the U-Net predictions in the most-challenging conditions is given in Figure 3. As the network always retrieves the correct answer when the cylinders do not intersect, Figure 3 shows the results for four samples randomly selected among the subset of samples wherein the targets are nested. Consistent with the performance metrics, the framework appears to be capable of retrieving both targets with high accuracy, even if one of the targets has a high contrast. In doing so, the most critical pixels are of course those at the shared boundaries, where the OSM image may bias the result, as for instance happens for the sample in the third row, where the network assigns the round shape to the stronger scatterer, in agreement with the OSM reconstruction at 8 GHz.

## 7. Validation on Experimental Data: The Fresnel Database

**Figure 4:**
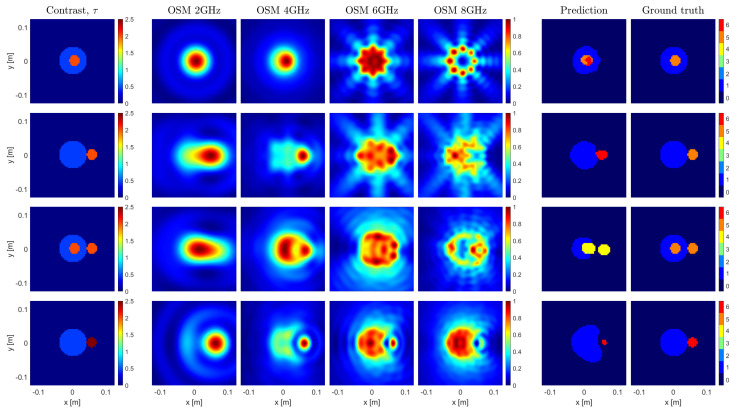
Imaging results of 4 experimental cases. The first column depicts the contrast of the imaged samples. The following 4 columns represent the OSM indicators at 2, 4, 6, and 8 GHz. The 6th and 7th column show the predictions made by the U–Net and their corresponding ground truths, respectively.

The proposed framework, as implemented and trained in Section 5, was validated with the experimental data provided by the Fresnel Institute of Marseille [26], which represents the commonly adopted benchmark for MWI methods. The targets of this database are depicted in the first column of Figure 4.

The Fresnel database includes cases and conditions that are substantially different with respect to those considered so far. In particular, the dielectric materials employed to build all the targets are not exactly lossless, and no sample in the training set exactly corresponds to Targets 1 and 2. Moreover, the third case is made of three cylinders, whereas only two cylinders were considered in the training. Last but not least, the fourth case has a metallic target, while only dielectric targets were generated for the training. Finally, the Fresnel targets were measured under an aspect-limited configuration, which poses a further difficulty, as that lack of data may lead to a worse OSM estimate.

Despite the above, the assessment was carried out without performing a specific training to accommodate the Fresnel measurement configuration and targets, but using the network resulting from the training described in Section 5. In this way, a proof-of-concept of the flexibility and robustness of the proposed approach is provided. As a matter of fact, while the OSM can handle a broad variety of targets and measurement configurations without changing the size in pixels of the resulting images, the OSM imaging results are of course affected by these different targets and measurement conditions. Hence, the images the OSM provides to the U-Net when processing Fresnel data are different from those the network was trained with in Section 5, thus making this validation a challenging and cogent test.

The obtained results are reported in the sixth column of Figure 4, while the seventh column reports the nominal ground truth. As can be seen, despite the aforementioned difficulties, the targets were quite successfully retrieved. More in detail:

- In the first three cases, it appears that there is an inherent difficulty for the network in differentiating the two upper contrast classes. This is a consequence of the reported 10% measurement error in the high-contrast target of the experimental data [26]. As a matter of fact, such and error makes the small targets fit into three of the segmented classes.
- The network was able to process the third sample even though it was not trained to identify and process samples with three targets. Nevertheless, it retrieved all the targets, but for a slight shape degradation of the low-contrast target. This is a consequence of the fact the U-Net performs a pixelwise classification.
- The presence of the metallic target in the fourth sample significantly affects the spatial distribution of the OSM indicator. This influences the U-Net prediction of the unknown targets’ shapes. However, the class assignation for both targets is correct. In particular, the metallic target was allocated to the highest contrast class.
- Due to the above-mentioned uncertainty on the EM target’s properties and the fact the position of the targets can be slightly different from the expected ground truth, the metrics for the experimental cases were not computed. However, the quality of the results can be assessed by comparison with the literature. For instance, References [37,38,39,40] reported some very recent DL-MWI methods, which were assessed with some targets taken from the Fresnel database. It is worth noting that, unlike those papers, the framework herein presented was not specifically retrained to deal with the Fresnel data, thanks to its inherent robustness and flexibility.

## 8. A Preliminary Example on Brain Stroke Imaging

Brain stroke imaging is one of the main perspective applications of MWI [1]. In this framework, it is crucial to discriminate between ischemic and hemorrhagic stroke, in order to administer the proper treatment in a timely manner [1]. Since ischemic tissue and hemorrhages have different electromagnetic properties, MWI represents a potential candidate technology, capable of providing a diagnosis in real-time and using a low-cost apparatus [1].

To show the potential of the proposed DL-MWI approach, a preliminary example of stroke classification from MWI raw data is presented in the following. To implement the framework, the selection of the segmentation classes was based on the specific application requirements. Accordingly, $N_{C}=3$ classes of contrast values are defined, one for each type of the stroke and one for the non-stroke tissue, i.e., the background. The network was trained with the OSM imaging results corresponding to the two types of stroke, for different sizes and positions within the brain. The training set was constituted by N=3000 samples. Among them, 90% were used as the training set and 10% as the test set.

Since, in this case, lossy media are involved, the framework has to be trained considering this condition. However, this does not represent a difficulty for the data simulation needed to build the training pairs, since the same approach to the solution of the forward problem as in Section 5 can be exploited. Furthermore, since OSM indicators encode the variation of the complex contrast, no change was needed in the U-Net architecture, since the inputs were similar to those treated so far. The MWI raw data were simulated with respect to the 3000 contrast maps built on the basis of a brain slice taken from the available tissue segmentation of the Zubal phantom [41], in which a stroke is inserted. For the sake of simplicity, the stroke was modeled as a circular cylinder, with the radius in the (1–2.6) cm range. Note that, in generating the samples, it was assumed that only one kind of stroke at the time was present and that at least a stroke was present in the inspected scenario. Accordingly, each sample of the dataset had at most two classes (background and one kind of stroke).

The dielectric properties of each tissue were assigned according to [42], in which tissue frequency dependence is based on a four-Cole–Cole model [43]. Moreover, tissues within the dataset were randomly perturbed with ±10% variability with respect to the values given in [42] to mimic inter-subject variability. For the ischemic and hemorrhagic strokes, the resulting amplitudes of the mean contrast values characterizing each class are reported in Table 3. The contrast values were computed with respect to the background medium.

For each brain slice contrast map, the MWI raw data were simulated using $N_{v}=N_{m}=16$ antennas and $N_{f}=4$ evenly spaced frequencies in the (0.5–1.25) GHz range and considering as a background a matching medium with a permittivity of 23 and conductivity of 0.19 S/m [1].

These data were finally used to generate the OSM indicators. In doing so, the Green’s function in (4) was replaced with the one pertaining to the healthy brain scenario, which was assumed to be known. Moreover, the OSM indicator was computed processing the differential data obtained by subtracting the healthy brain data from the stroke-affected ones.

**Figure 5:**
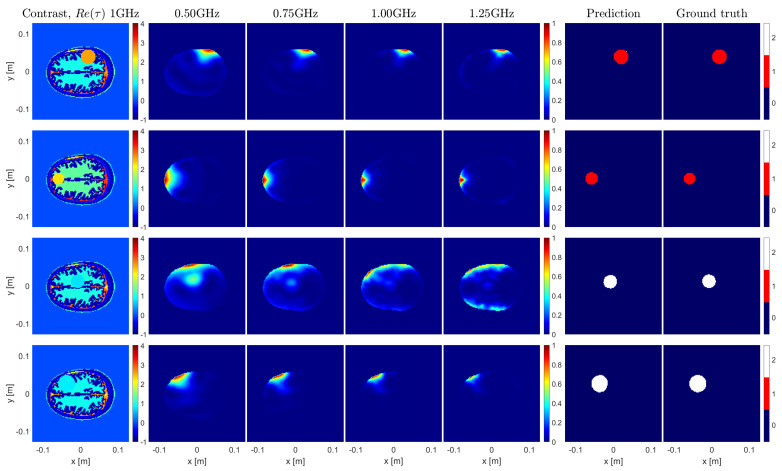
Sample results for the brain imaging example. The first column depicts the contrast of the imaged samples. The following 4 columns represent the OSM indicators at 0.5, 0.75, 1.00, and 1.25 GHz. The 6th and 7th columns show the predictions made by the U–Net and their corresponding ground truths, respectively.

For each sample of the test set, the considered metrics were calculated and averaged over 10 folds. Overall, performance scores above 87% were achieved for both metrics, as shown in Table 3. In Figure 5, some random samples from the test set are shown to give a visual idea of the obtained results and of the fact that the U-Net succeeded in extracting information that was not obvious at all in the OSM imaging results.

## 9. Conclusions

In this work, an innovative DL-MWI framework for an effective, reliable, and fully automatic solution of the ISP was presented. The proposed approach combines qualitative microwave imaging and deep learning in a synergistic framework, which exploits a qualitative imaging technique called the OSM jointly with a DL architecture called the U-Net. The former, though qualitative, provided an estimation of the contrast distribution. The latter managed to address the ill-posedness and non-linearity of the ISP to retrieve the electromagnetic properties of the targets. On top of that, both the OSM and U-Net are computationally efficient techniques, allowing, after the training stage, real-time imaging results.

The choice of the OSM was motivated by the fact that it allows automatic and real-time processing of MWI raw data gathered under different measurement conditions and from different targets. These features are important to make the pre-processing as robust as possible and allow the overall framework to be deployed in real-time, with no need for human supervision, and in different situations. In addition, the OSM image has also the added value of providing information on the spatial variations of the contrast. However, this information is implicit in the OSM image, meaning that there is no obvious connection between the OSM indicator value and the contrast value. The choice of U-Net then comes from the need to handle this difficulty: U-Nets have a proven capability of solving classification problems underlying complex models, and the proposed approach aims to take advantage of their capabilities. In particular, the problem was cast in terms of a segmentation task by training the U-Net to associate the pixels of the OSM imaging results to a finite set of contrast values. To the best of our knowledge, the proposed approach is the first one in which the concept of segmentation is exploited to solve the ISP.

A performance analysis of the processing framework was carried out on simulated data and quantitatively assessed by means of two metrics. The outcome of this analysis led to performance scores above 77% for both metrics, which may represent a satisfactory result, depending on the targeted application. In any case, performance scores can be further improved in several ways. A first and straightforward approach would consist of increasing the size of the training set, instead of using the criterion derived from [21]. A more subtle strategy would consist of implementing a different sampling scheme of the permittivity values, using a finer discretization for the higher permittivity range. In this respect, sampling the contrast range unevenly based on the different degree of nonlinearity pertaining to the contrast values of interest could be an interesting path to explore [36].

Validation results on Fresnel experimental samples were provided, showing the compelling capabilities of the proposed DL-MWI approach. In fact, some of those results were based on samples and conditions that were significantly different from the ones considered in the optimization of the network. The successful retrieval of outliers of the dataset demonstrates the generalization capability of the framework, also considering that the U-Net was not specifically retrained to handle the Fresnel data, as usually performed in the literature.

An example concerned with brain stroke imaging and classification was reported. While preliminary, the achieved results show the potential of the proposed approach when dealing with complex scenarios relevant to applications and call for a more extensive assessment considering a larger set of cases and conditions, as well as limiting or avoiding the need for a priori information on the healthy brain.

While the above-described results support the validity of the proposed approach and its flexibility in handling different conditions, it must be remarked that the quality of the OSM imaging results is influenced by the measurement configuration. On the other hand, the implications of the low quality of the OSM images on the overall results are not straightforward, since simply increasing the size of the training set could counteract it. Would this not be possible or effective, then a possible way to overcome such a limitation can be to train the network with the imaging results obtained from OSM along with those obtained from other MWI approaches. Of course, the choice of the proper MWI approach to exploit would be dictated by the specific measurement configuration at hand and the application.

A very important aspect that deserves further investigation is the choice of the neural network architecture. In this paper, the U-Net is a “natural” choice since this network was designed to perform segmentation tasks [18] and the aim of the work was to prove the feasibility of the DL-MWI framework. However, several architectures have been developed in the last few years [44], which are worth being considered to possibly improve the performance of the approach to solve the ISP, as well as to handle specific scenarios more effectively. A first option is the enhanced version of the U-Net, U-Net++, which is a deeply supervised encoder–decoder network that achieves improved segmentation results [45]. The attention U-Net [46], making use of an attention gate to trim features that are not relevant to the ongoing task, can be instead exploited when the targets of interest are localized and the surrounding is not relevant, such as in monitoring tasks. Finally, to handle scenarios in which features at different scales are relevant, the MDC-Net or feature pyramids with the U-Net++ could be considered to accommodate multi-scale segmentation or feature extraction [47,48].

Finally, the approach was herein developed and presented for the 2D scalar ISP, which is appropriate to demonstrate the validity of a novel approach and useful in many cases. However, it is important to pursue the extension to the 3D geometry, which is both more challenging and important for applications. While this extension is not straightforward from a computational point of view, it is worth noting that all the “building blocks” are available, since 3D U-Net architectures have been already implemented [49] and the 3D version of the OSM has been recently developed [50].

## Figures and Tables

**Table 1:** Simulations for training data generation.

Size of imaging domain	25×25 cm^2^
Image size in pixels	64×64
Pixel size	0.1526 cm^2^
Background medium	Air
Number of illuminating antennas $N_{v}$	8
Angular spacing between emitters	45°
Number of receiving antennas $N_{m}$	241
Angular spacing between receivers	3/2°
Distance of the source from the center of Ω	167 cm
Distance of the receiver from the center of Ω	167 cm
Number of frequencies $N_{f}$	8
Frequency range	[2,9] GHz
Frequency step	1 GHz
Target radius range	[1.2,5] cm
Maximum target electrical size	1.3λ
Minimum target electrical size	0.08λ
Target relative permittivity range	[1.3,3.5]

**Table 2:** Ten-fold cross-validation averaged performance for the different segmentation classes.

Segmented Classes and Color Label	Contrast τ	*DSC*	*MCC*
6	(2.2,2.5]	0.890	0.797
5	(1.8,2.2]	0.873	0.798
4	(1.4,1.8]	0.867	0.777
3	(1.0,1.4]	0.841	0.801
2	(0.6,1.0]	0.872	0.813
1	(0,0.6]	0.886	0.853
0	0	0.998	0.979

**Table 3:** Performance of the DL-MWI approach for stroke imaging and classification, split by kind of tissue.

Segmented Classes and Color Label	Contrast Amplitude Range 1 GHz	DSC	MCC
Ischemia	0.96±10%	0.861	0.876
Hemorrhagic	2.33±10%	0.936	0.923
Background	0.0±10%	0.999	0.918
